# Corrosion suppression and strengthening of the Al-10Zn alloy by adding silica nanorods

**DOI:** 10.1038/s41598-024-64323-x

**Published:** 2024-07-08

**Authors:** Eman AbdElRhiem, Yosry F. Barakat, Shereen M. Abdelaziz, M. M. Mostafa, R. H. Nada, Saad G. Mohamed

**Affiliations:** 1 Mining and Metallurgy Engineering Department, Tabbin Institute for Metallurgical Studies (TIMS), Tabbin, Helwan 109, Cairo, 11421 Egypt; 2 Physics Department, Faculty of Education, Ain Shams University, Heliopolis, Roxy, P.O. Box 5101, Cairo, 11771 Egypt

**Keywords:** Al-Zn alloy, Nano-silica addition, Mechanical properties, Corrosion resistance, Grain refining, Chemistry, Materials science, Physics

## Abstract

Aluminum alloys have been widely studied because of their current engineering applications. Due to their high strength and lightweight, cracking can easily initiate on their surface, deteriorating their overall functional and structural properties and causing environmental attacks. The current study highlights the significant influence of incorporating 1 wt% silica nanostructure in aluminum-10 zinc alloys. The characteristics of the composites were examined using Vickers hardness, tensile, and electrochemical testing (OCP, Tafel, and EIS) at various artificial aging temperatures (423, 443, and 463 K). Silica nanorods may achieve ultrafine grains, increase hardness by up to 13.8%, increase σ_UTS_ values by up to 79% at 443 K, and improve corrosion rate by up to 89.4%, surpassing Al-10 Zn bulk metallics. We demonstrate that silica nanorods contribute to the creation of a superior nanocomposite that not only limits failure events under loading but also resists corrosion. Our findings suggest that silica nanocomposite can produce unique features for use in a variety of automotive, construction, and aerospace applications. This improvement can be attributed mainly to the large surface area of nano-silica particles, which alters the Al matrix. Microstructural, mechanical, and electrochemical studies revealed that the effects of structure refinement were dependent on nano-silica.

## Introduction

Al-Zn alloys were initially designed for lightweight military bridges, but they are currently used in commercial marine engineering applications, ship transportation, and rail transit systems, among others, and are commonly utilized in welding applications^1–3^. Their usage has been limited elsewhere because of fears of stress-corrosion cracking along welds. Furthermore, aluminum alloys have been proven to suffer from oxide formation. Due to the challenges related to component manufacturing, researchers have explored many solutions, including parameter optimization, alteration of alloy composition^4–6^, and post-processing techniques. These approaches aim to address faults and enhance the production of components with superior mechanical properties^7^. Since further studies focus on aluminum alloys with many destructive configurations, only a few of them address the effects of heat treatments on structures^8^. The aging of aluminum-Zn alloy offers the greatest potential to enhance the strength properties required for aerospace structures^9^ by precipitating particles during the supersaturated solid solution transformation. Another intriguing concept is to use nanostructures as reinforcing materials. A variety of reinforcements are used to improve the properties of matrix materials, including SiO_2,_ TiO_2_, CuO, Al_2_O_3_, SiC^10^ boron carbide, titanium carbide, titanium diboride, silicon nitride, silicon carbide, and zirconium dioxide^11,12^. Nanoparticulate-reinforced materials serve as nucleation sites for solidification, resulting in enhanced durability and high strength and an improvement in both yield strength and ductility. Karakoç et al. and Karabulut et al.^13,14^ both observed and reported on compacted reinforced aluminum, corrosion-proof (verification) structures for aircraft, ships, vehicle buildings, and automobile components such as drive shafts, cylinders, pistons, brake rotors, and finer-grain industrial alloys^15,16^.

SiO_2_ particles are used commercially in the manufacturing of Al-based composites due to their superior mechanical and physical properties^17^. Increasing its characteristics has been challenging due to their applications, including high surface area, well-defined surface properties, chemical, thermal, and water stability, size and shape tunability, and high loading capability^18^. A previous study^9,10^ indicates that the Al-10 Zn alloy gains microhardness and misconstrues characteristics when ceramic particles' of TiO_2_, SiO_2_, and CuO are added. The presence of CuO nanoparticles enhances the corrosion resistance of the Al-10 Zn alloy. The addition of SiO_2_ nanoparticles to the aluminum matrix comes with other problems, such as the formation of agglomerates and poor dispersion^19^. Issa et al.^20^ produced Al-SiO_2_ nanocomposites using hot extrusion and powder metallurgy, improving mechanical properties and increasing hardness and tensile strength by 41.8% and 24.8%, respectively. An increase in silica concentration may result in a reduction in its tensile properties^20^. Zhu et al.^21^ fabricated an Al-Si/α-Al_2_O_3_ composite by including 30 wt% SiO_2_ micro-particles using exothermic dispersive synthesis employing mixes of silica and Al powder. Huo et al.^22^ investigated in situ fabrication using ball milling to obtain fine particles of silica and aluminum. Compared to other reinforcing materials, silicon dioxide is used more frequently to improve structural properties. Aluminum-based nanocomposites are manufactured using various techniques, including powder metallurgy, centrifugal casting, additive manufacturing, and stir casting^12^. Stir casting is the simplest, least expensive, and most straightforward method of making composites^23^. Bahri et al.^24^ investigated the corrosion behavior of nano-silica potassium silicate that was coated on the 2024 aluminum alloy; the presence of nano-silica improved the silicate networks and decreased the existing defects. The ratio of nano-silica played an important role in providing high corrosion protection. The addition of small dosages of nano-silica caused a delay in microstructure development and durability improvement in concrete.

A literature survey revealed that no research on mechanical and electrochemical applications incorporating nano-sized SiO_2_ particles had been published, and few studies had been conducted. Therefore, this study seeks to present the dominant rule behind the use of silica nanostructure in Al-Zn alloys, as well as the present state of execution and outcomes of nanostructure-modified alloys as a reinforcement to enhance the alloy’s strengthened (mechanical), electrochemical, and microstructure characteristics, and highlights the importance of the aging process to achieve a highly superior nanocomposite, making it more suitable for various applications in industries such as aerospace and automotive. The samples are subjected to an investigation at room temperature to analyze the mechanical and electrochemical properties of the material after aging the samples for two hours at different temperatures (423, 443, and 463 K).

## Materials and methods

### Composites fabrication

Al-10 wt% Zn alloy (AZ) was prepared by melting high-purity components in a vacuum. The castings were cut into dimensions of 16 × 80 × 200 mm^3^. 1 wt% of commercial silica nanostructure powder with nano-sized (10–80 nm) and 99.8% purity was provided by Alfa Chemistry Manufacturing Company. Nano-silica was added to the base AZ alloy after being remelted mechanically in a vacuum furnace at 1000 K to prepare the nanocomposite (AZS), solution-treated at 500 °C for 2 h, and quenched in cold water. Samples (AZ, AZS) were swaged and cold-drawn into sheets with dimensions of 10 × 10 × 1 mm^3^. The samples were solution-treated (solidification) at 500 °C for 2 h and quenched in cold water to remove contaminants from the cold working process. Following artificial aging, the samples are cast and homogenized before being used. Aging steps took place for 2 h at different temperatures ranging from 423 to 463 K, then quenched with cold water to preserve the structure.

The chemical composition of the composites was examined using X-ray photoelectron spectroscopy (XPS, Thermo Scientific).

The chemical composition weight percent (wt%) of the investigated composites is shown in Table 1.

**Table 1:** Chemical composition of the composites by weight percentage (wt%).

Sample	Symbol	Zn	Cu	Ti	Si	O	Al
Al-10 Zn	(AZ)	9.58	–	–	–	–	Bal.
Al-10 Zn-1SiO_2_	(AZS)	9.5	–	–	0.6	0.35	Bal.

### Composites characterization

Mechanical, metallurgical, and electrochemical tests were performed on the manufactured Al-10Zn and Al-10Zn-1SiO_2_ composites to investigate their properties.

Optical Microscope (OM), the microstructure investigations of samples were carried out using a quantitative image analyzer microscope of the type (Leco Lx 31) for an obvious microstructure; Keller’s reagent etching was used to show the orientations and boundaries of the grains. The solution consisted of 95 ml of distilled water, 2.5 ml of HNO_3_, 1.5 ml of HCl, and 1 ml of HF, and then the samples were cleaned with water and alcohol before being dried using hot air.

Scanning electron microscope (SEM) analysis was performed on a number of sheet specimens to identify structures and study surface changes that happened before and after various aging temperatures.

Transmission electron microscopy (TEM) images of nanostructured SiO_2_ powders were taken with a model JEOL JEM_2100, indicating that the added materials are nanoscale.

XRD patterns can reveal the crystal’s interior structure. The phases present in the raw material were identified using the X-ray diffraction technique. An X-ray diffraction pattern was generated using a Panalytical X’pert BRO XRD unit. A copper cathode produces X-ray radiation with a wavelength of 0.154 nm. An incidence angle of 2^0^ corresponds to an X-ray penetration depth in the sample. Diffractograms of the samples were produced.

Tensile properties were acquired by Beijing Sinofounf Co., Ltd. (China), a computer-controlled universal testing machine, model WDW-300. Testing is also done at 0.1 mm/min crosshead speed. The tensile samples are wire samples that have an initial length of 300 mm and a diameter of 7 mm. To determine their tensile properties, the original cross-sectional area and a benchmark measuring 2 mm were utilized under the effect of different aging temperatures within the range of 423–463 K. The computer automatically computed the tensile strength and elongation at break at room temperature. For every sample, the mean of a minimum of four measurements was provided. The experimental error was ± 10%.

Microhardness was measured using a Vickers microindenter apparatus of type Leco LM70 under a constant load of 300 g for 10 s. The examined samples were heated in 20 K increments from 423 to 463 K. Following that, the composites’ surfaces were polished, and microhardness tests were performed at room temperature to ensure that the structures formed during the aging process remained stable. The average of each sample was calculated using at least four measurements. The sample’s surface was examined under an HMV^−2^ measuring microscope with a magnification of 50×. The experimental error was ± 10%.

**Figure 1:**
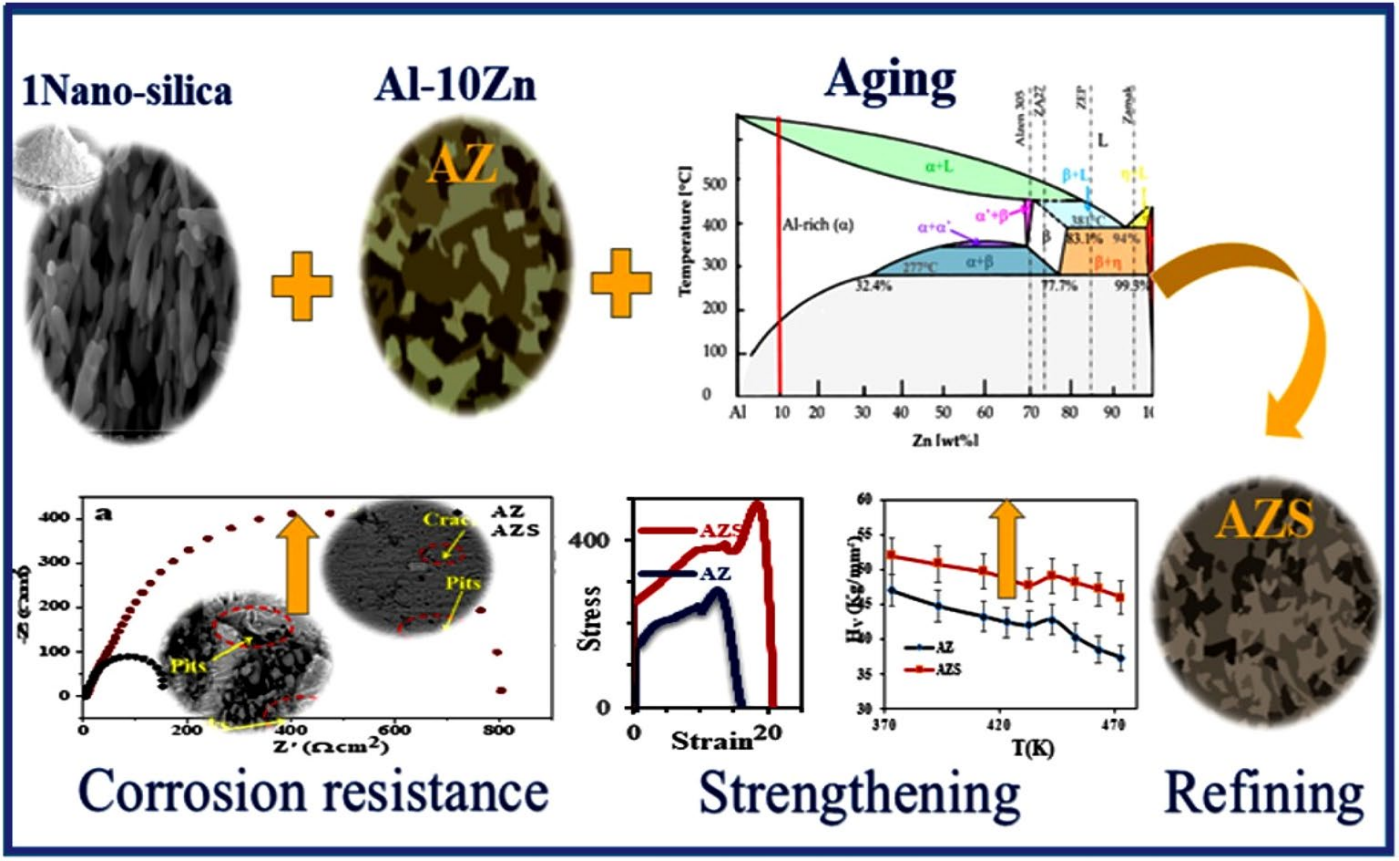
A diagrammatic illustration of the Al–10Zn and Al-10 Zn-1SiO_2_ investigations.

An electrochemical testing station (Origafex-OGF01A-Origalys, France) was used to determine the corrosion rate at room temperature using a three-electrode system according to ASTM G59-97. After being cleaned with deionized water, the composites were dried and cleaned ultrasonically in acetone. Then, a solution of 3.5% NaCl was used for the corrosion test. The reference electrode was an Ag/AgCl, and the auxiliary electrode was a Pt sheet. The electrochemical impedance spectroscopy measurements (EIS) were executed at the open circuit potential. Tafel were used to calculate the corrosion rate (C.R.) and corrosion current density (I_corr._) to determine corrosion behavior at a scan rate of 2 mV/s and a potential range of -300 to 300 mV over 30 min. The measurements were performed in a frequency range of 0.1 Hz to 100 kHz with an amplitude of 10 mV, as quantified by ASTM G106-89. Figure 1 shows the systematic diagram for the full investigations, which comprises mechanical, electrochemical, and microstructure analyses of the Al-10Zn and Al-10Zn-1SiO_2_.

## Results and discussion

### Structural characterizations

#### Microstructure of the silica

**Figure 2:**
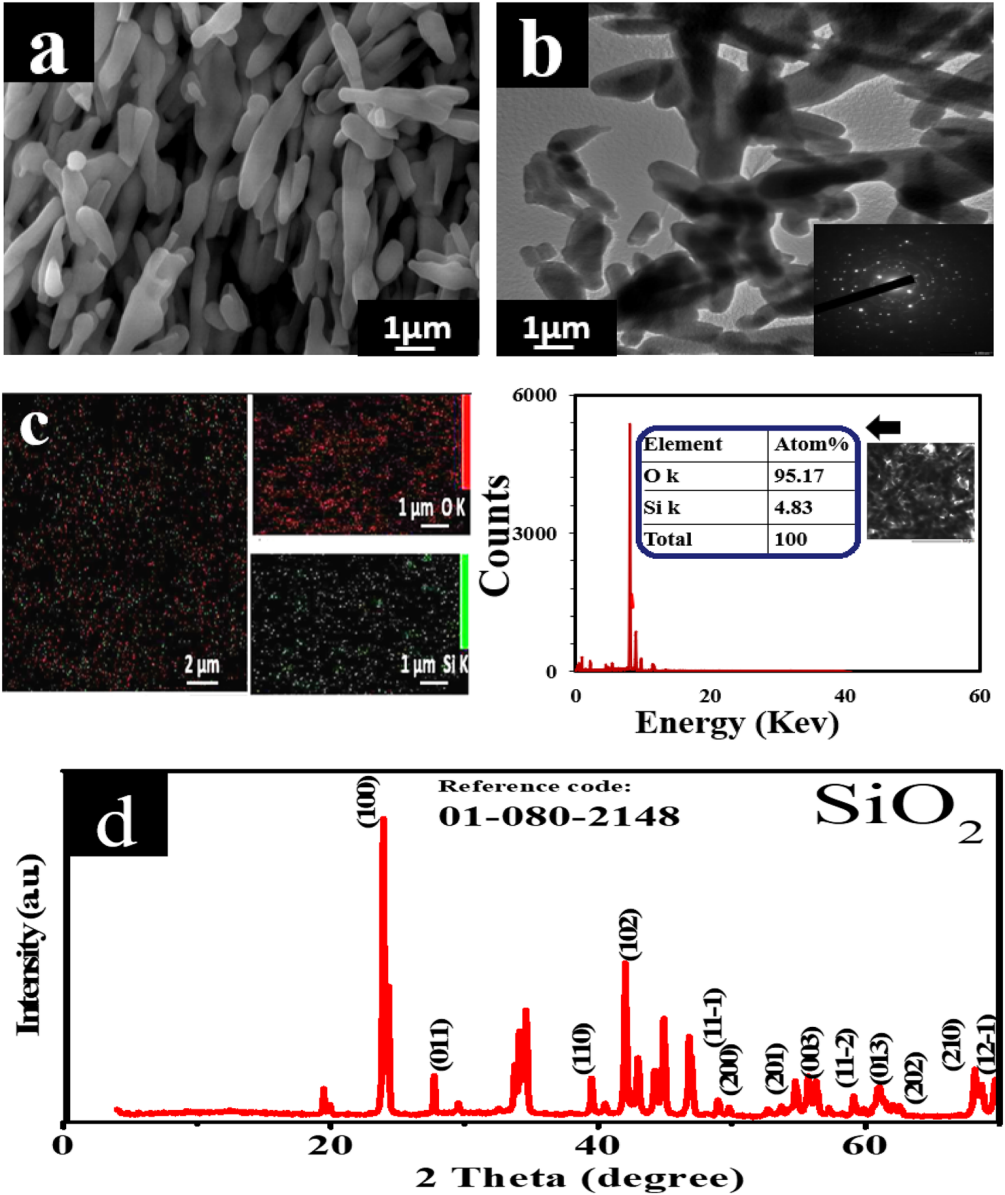
(**a**) SEM, (**b**) TEM, (**c**) EDX images, and (**d**) XRD pattern of nano-silica.

The crystal structure and microstructure examination of the nanostructure (NS) were acquired with SEM, TEM, EDX, and XRD analysis. SEM, and TEM images in Fig. 2a,b show the morphologies of SiO_2_, which appear in the form of nanorods. Figure 2a and b indicate that the NS at the nanoscale is in the range of 20–80 nm. Figure 2c shows the EDX analysis of SiO_2,_ which confirms the chemical compositions and indicates that the SiO_2_ nanoparticles have no impurities. The XRD pattern of silicon dioxide is depicted in Fig. 2d, showing a hexagonal SiO_2_ phase (card no. 01-080-2148). The XRD patterns display sharp peaks, showing that the nanostructured powders used are very crystallized^25^.

#### Composite structural

**Figure 3:**
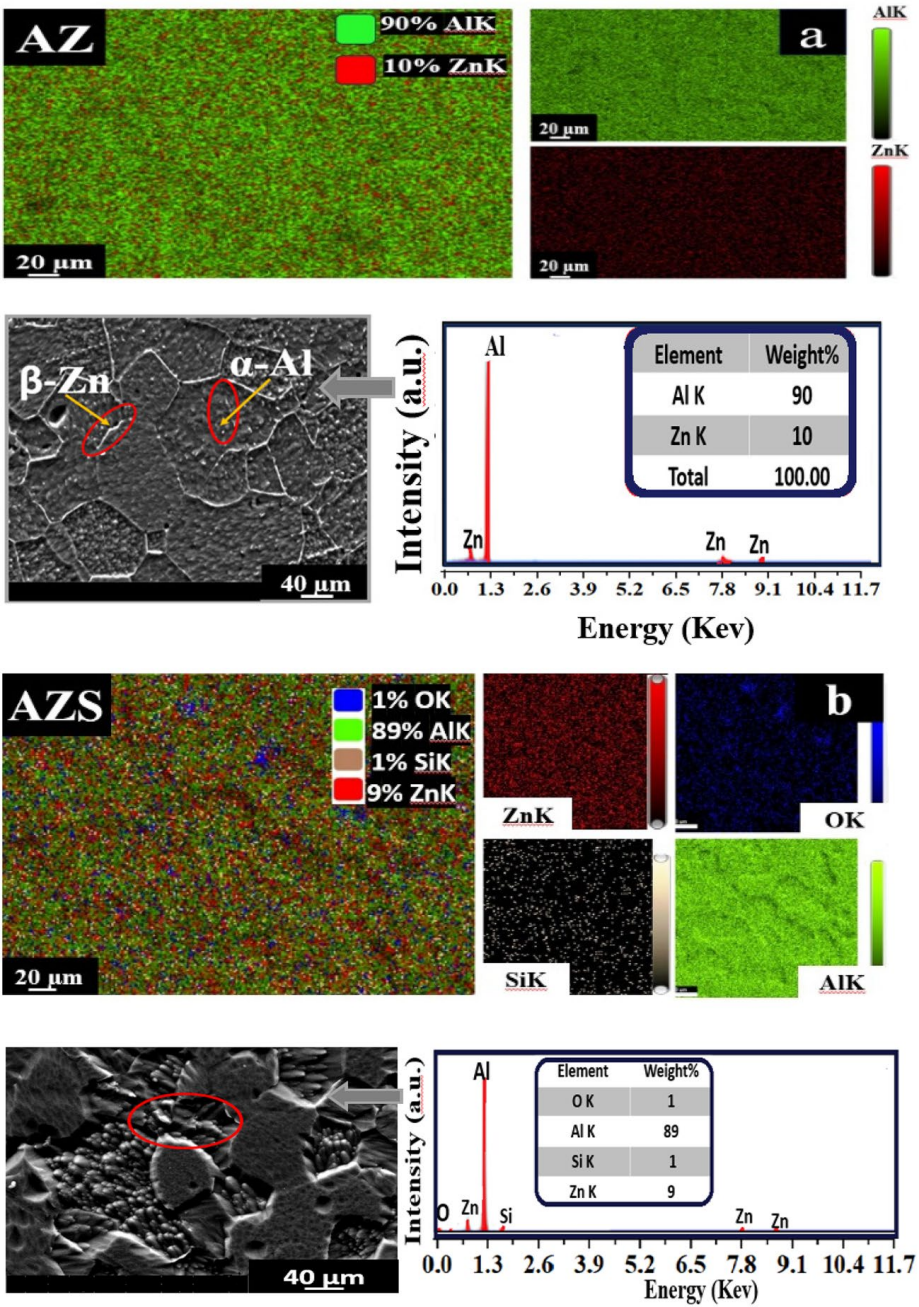
EDX mapping images, SEM images, and EDX analysis of (**a**) AZ (base alloy) and (**b**) AZS composite.

SEM images reveal a uniform microstructure consisting of two distinct metallurgical phases: the α-Al matrix and the β-Zn phase. In Fig. 3a, the grain boundary contains nucleated Zn particles, which appear as big particles with a light gray color. The dark gray color indicates Al grains. The silica NS, when combined with the base alloy, forms the aggregated white particles^9,10^, as seen in Fig. 3b. The EDX mapping analyzes the chemical compositions of samples AZ and AZS. Moreover, Fig. 3b shows the important efficiency of the silica nanorods in terms of dispersion. Zinc has a tendency to precipitate at the interfaces between grains. This increases the probability of nanorods adhering to each other on the Zn particles, resulting in the formation of brilliant gray precipitates at the outer boundaries of the grains, as seen in Fig. 3b. This phenomenon takes place throughout the process of aging and results in a significant enhancement in the strength of the samples. The zinc deposits serve as nucleation sites for the process of solidification, leading to the formation of a smaller grain size^9,10,26^.

#### Effect of NS addition

**Figure 4:**
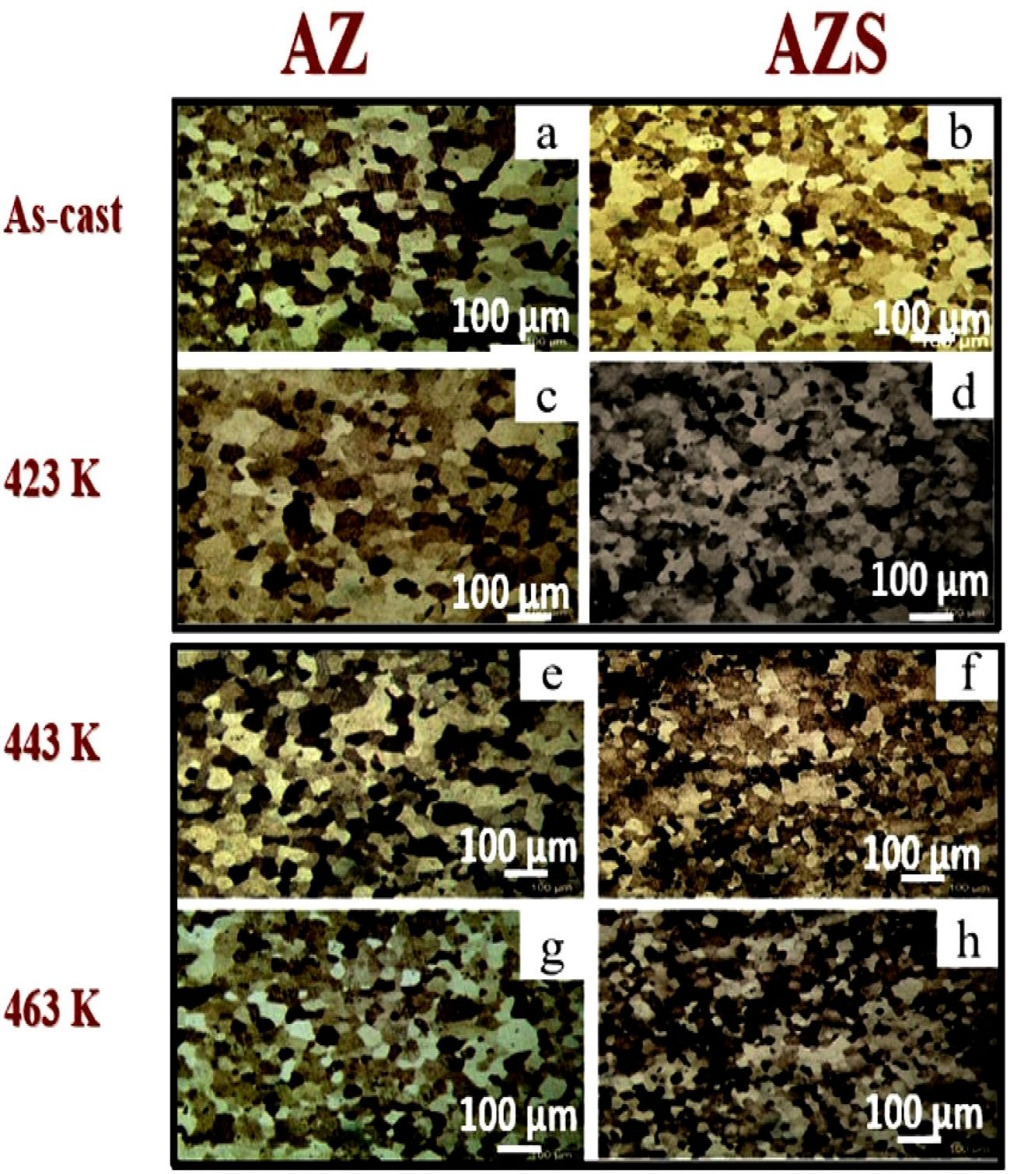
Typical optical images of AZ and AZS composites before and after aging temperatures (423, 443, and 463 K) with a scale bar of 100 µm.

Figure 4a–h shows optical micrographs (OM) of AZ (base alloy) and AZS nanocomposite before and after aging at temperatures (423, 443, and 463 K). Figure 4a,c,e, and g represent the as-cast AZ base alloy and the samples aged at 423, 443, and 463 K, respectively. Figure 4b,d,f,h show the corresponding AZS sample. Adding SiO_2_ NSs to the Al-10 Zn alloy and increasing the aging caused grain refinement^10,27^.

Grain size estimates using the Image J software indicated that fine-refining takes place at all aging temperatures in comparison to the as-cast condition, as shown in Table 2. The structure of all aged samples was modified by aging, resulting in a more uniform distribution of solutes (Zn and NS), especially in AZS compared to the AZ base alloy^9,10,27^. The grain orientations of the AZS nanocomposite differ from those of the AZ base alloy, as seen in Fig. 3. This increased dislocation density of the AZS nanocomposite, where the SiO_2_ NSs interact with the metal matrix and improve the surface areas, resulting in fine graining and structural modification^28^ before and during aging temperatures^27^, where AZS caused the most refining. The average grain size for AZ is 110 μm, whereas AZS is 93 μm. As a result, aging altered the structure of the aged samples due to the more uniform distribution^29^ of NSs, particularly for AZS rather than AZ.

**Table 2:** The grain size calculations of the Al grains of the composites before and after aging temperatures (423, 443, and 463 K).

Grain size (µm)				
Sample	As-cast	423 K	443 K	463 K
AZ	190	102	77	90
AZS	170	80	59	65

The effect of grain size on mechanical and electrochemical properties can be considered from two perspectives: (i) grain boundaries may act as obstacles to dislocation motion, resulting in a strengthening effect in a matrix of small grain sizes, and (ii) the presence of nano-sized particles acts as pinners to grain boundary migration, improving its properties^27^. Aging temperature is an important consideration in refining both composite microstructures^9,27,30^. In addition, the retardation effect of silica nanorod addition is superior to that of AZ^28^.

### Mechanical tests

#### Tensile tests

**Figure 5:**
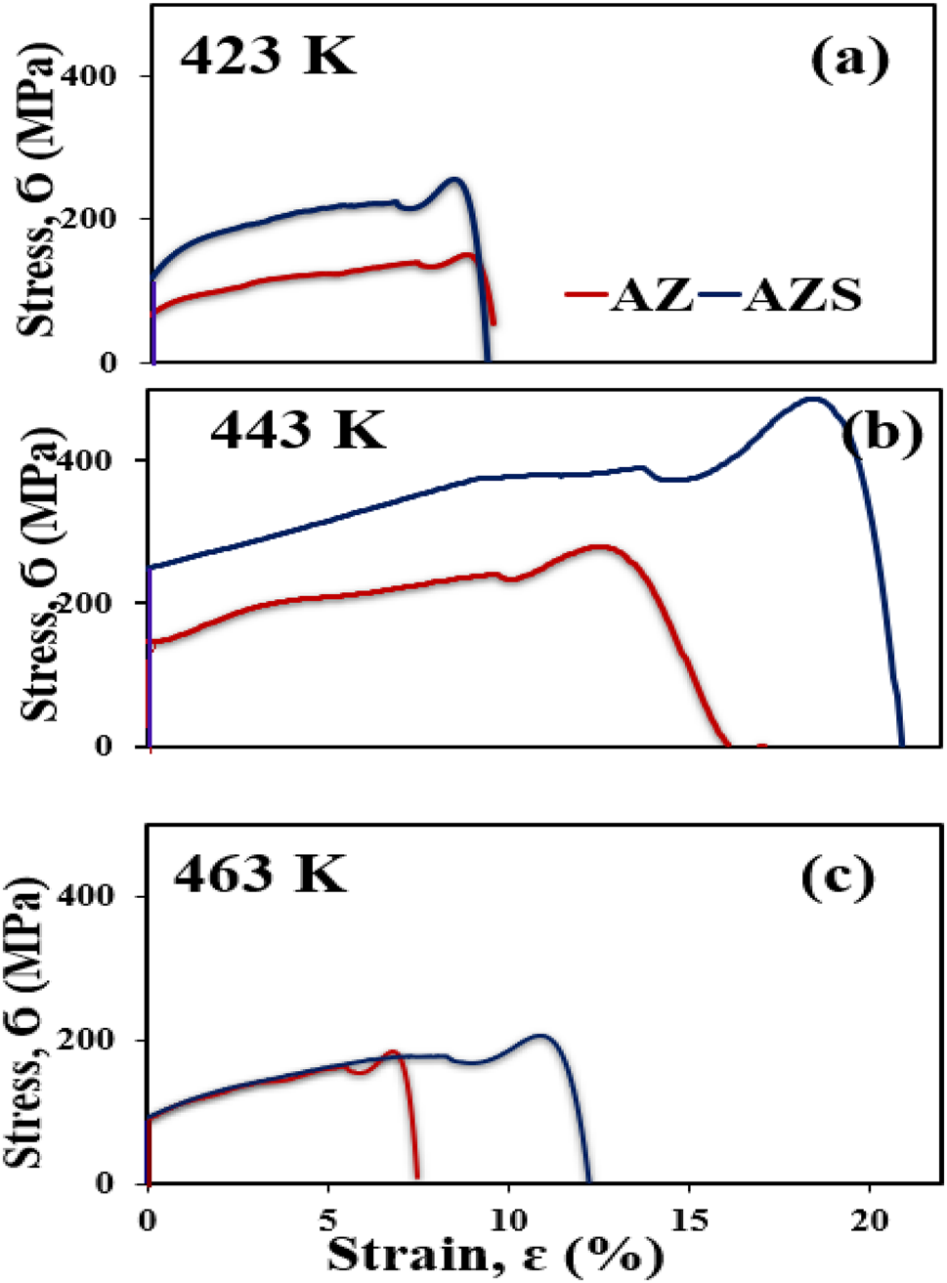
Stress–strain curves for AZ and AZS at (**a**) 423 K, (**b**) 443 K, and (**c**) 463 K at a constant strain rate of 2.5 mm/min for AZ and AZS composites.

Figure 5a–c shows the results of systematic tensile tests conducted for the two composites (AZ and AZS) at a testing temperature of 25 °C (room temperature, RT) at a constant strain rate of ~ 2.5. Nanostructured metals exhibit high plasticity but low ductility; therefore, enhancing ductility involves increasing the strain hardening rate through nanostructure engineering, which is accelerated by crystalline defects (dislocations). Strain rate sensitivity may significantly improve the ductility of metals^31^. Aging plays a fundamental role in enhancing the ductility and plasticity of both composites^32^. Tensile stress–strain curves for AZ and AZS composites stretched at different aging temperatures within the range of 423–463 K. It is evident that for both composites, rising aging temperatures from 423 to 443 K caused the shifts of level curves in the direction of higher stress values, but at 463 K, there is a drop in ultimate and yield stress, as shown in Table 3. Figure 5 illustrates how the inclusion of silica nanorods affects the tensile characteristics. It reveals the relationship between strain and tensile stress at different aging temperatures (423, 443, and 463 K) for both composites. This graph demonstrates how increasing strain rates increased the σ_UTS_ in both composites. This is because a rising strain rate leads to an increase in dislocation density^33^. Table 3 illustrates the values of σ _UTS_, σ_y_, and σ_Frature_ for the composites at different aging temperatures.

**Table 3:** σ_UTS_, σ_y_, and σ_Frature_, of the composites.

423 K				443 K			463 K		
Sample	σ_UTS_ _MPa_	σ_y_ _MPa_	σ_Fracture_ _MPa_	σ_UTS_ _MPa_	σ_y_ _MPa_	σ_Fracture_ _MPa_	σ_UTS_ _MPa_	σ_y_ _MPa_	σ_Fracture_ _MPa_
AZ	151.8	135.4	139	278	238	263.9	177	166	177.6
AZS	250	218.7	241.5	482.6	391	472.9	204.9	174.5	186.5

As the dislocations migrate, they become mixed. Other dislocations make it more difficult to glide through the material, especially at lower temperatures. At high testing temperatures, dislocation annihilation appears to occur faster than dislocation creation during deformation. At higher temperatures, decreased strain rates resulted in lower strength in composites and dynamic recovery, leading to a drop in σ_UTS_ (Fig. 5)^34^.

Silica nanorod addition was shown to impact σ_UTS_ and σ_y_ values significantly. The increase in σ_UTS_ and σ_y_ values with rising aging temperatures and/or maximizing strain rate may be ascribed and understood by considering that the plastic deformation is initiated by the thermal effect and rate dependent on the stress itself^32,34^. The above interpretation, as described in our previous work^10^, finding nano-size SiO_2_ particles in the Al-Zn base matrix decreases the interfacial energy and consequently suppresses the growth of the grains in the Al-Zn matrix by increasing the dislocation density locations that accumulate at the grain boundaries by increasing the aging temperature, which causes a significant change in the microstructure that will lead to improved ductility compared with AZ composite. The age-hardening of the composites is analyzed up to large strains. Meanwhile, the ductility of two of the composites falls into the trend of previous experiments with increasing aging temperatures^34^. As shown in Fig. 5a and b (at 423 and 443 K for AZ and AZS nanocomposite), it stands out by combining high strength with high ductility. The excellent properties of 443 K, as shown in Table 3 are assumed to be due to a favorable size distribution of nano-silica in the Al-Zn base alloy and the completely dissolved Zn in the Al matrix (phase transition), where nano-sized SiO_2_ particles pinning the grain boundaries result in ultrafine grains^27,28,30^, which confirm the hardness measurement. Increasing the aging temperatures to 463 K, as shown in Fig. 5c, decreases the strain to failure linearly with increasing yield stress. Increasing the strain percentage confirms the coarse grains produced using severe plastic deformation methods, as shown in Fig. 4, and the strong dispersion efficiency of nano-silica in the matrix, as shown in Figs. 2 and 3, respectively.

Raising the aging temperature to 463 K increased dislocation mobility (reducing dislocation density) without increasing grain size and is predicted to increase strain hardening and improve ductility and the available slip system^34^, resulting in, as seen in Fig. 5c, the AZ and AZS becoming soft and ductile with a small elastic area compared with Fig. 5b. Due to the phase transformation, the complete Zn dissolved in the Al matrix results in a decrease in σ_UTS_ and σ_y_ for the AZ composite, but the AZS composite is still superior to AZ. From this perspective, the obtained microstructure of the AZ and AZS composites can reflect the effect of silica nanorod addition on the tensile response and improve the tensile strength of the composite^24,28^, as well as the important rule of the impact of the aging temperature on the stress–strain characteristics^32^, and the higher durability of the AZS composite over the AZ.

#### Microhardness tests

The influence of nano-silica on the microhardness of the Al-Zn alloy is illustrated in Table 4. Under a constant load of 300 g, Table 4. illustrates the change in hardness values (H_V_) for both composites as a function of aging temperature (T) and dwell time (t) of 10 s.

**Table 4:** Hardness values of the composites under a 300 g load and a dwell time (t) of 10 s at different aging temperatures.

H_V_ (kg/mm^2^)									
Sample	373 K	393 K	413 K	423 K	433 K	443 K	453 K	463 K	473 K
AZ	47	44.8	43.3	42.4	42	42.8	40.3	38.5	37.3
AZS	52	50.8	49.7	48.7	47.8	49.1	48.1	47.2	46

The formula for the hardness number is^35^.

1 $${{\text{H}}_{\text{V}}}= 1.854\frac{\text{P}}{{d}^{2}}$$

where P is the applied load in N, d is the impression’s mean diagonal length in mm. H_V_ values decreased with increasing aging temperature, with a significant rise at 443 K (transition temperature) for AZ and AZS. AZS considerably enhances strength^10,28^ compared with AZ. At 443 K, anomalous behavior appeared as a peak, which indicates an increase in the hardness value due to favorable nano-silica size distribution in the AZ base alloy, which contributes to pin the grain boundaries^30,31^ and Zn’s complete dissolution in the Al matrix (phase transition), producing refining grains that validate the tensile measurements. Aging temperature increases zinc migration to grain boundaries, demonstrating strong dispersion. AZS (reinforced) seems to exhibit stronger evidence of dispersion strengthening due to its finer particle sizes than AZ (reinforced). This implies that a more efficient dispersion- strengthening mechanism is facilitated by the addition of finer particle sizes in AZS. Additionally, the reinforced AZS’s improved dispersion could result in better mechanical behavior^10,20,24,31^.

Dislocation interaction between Zn atoms is reduced when Zn dissolves, leading to a slight increase in grain size, a decrease in dislocation density, and a reduction in lattice strain. The composites (H_v_) decreased as a result of the decline in dislocation pinning activity^30^. Greater dislocation mobility as a result of the dissolving process caused a small increase in grain size and a corresponding decrease in lattice strain and dislocation density^10,30^. It should also be noted that ultrafine-grained metals processed using severe plastic deformation techniques frequently have a high density of dislocations.

### Electrochemical tests

#### Corrosion tests

**Figure 6:**
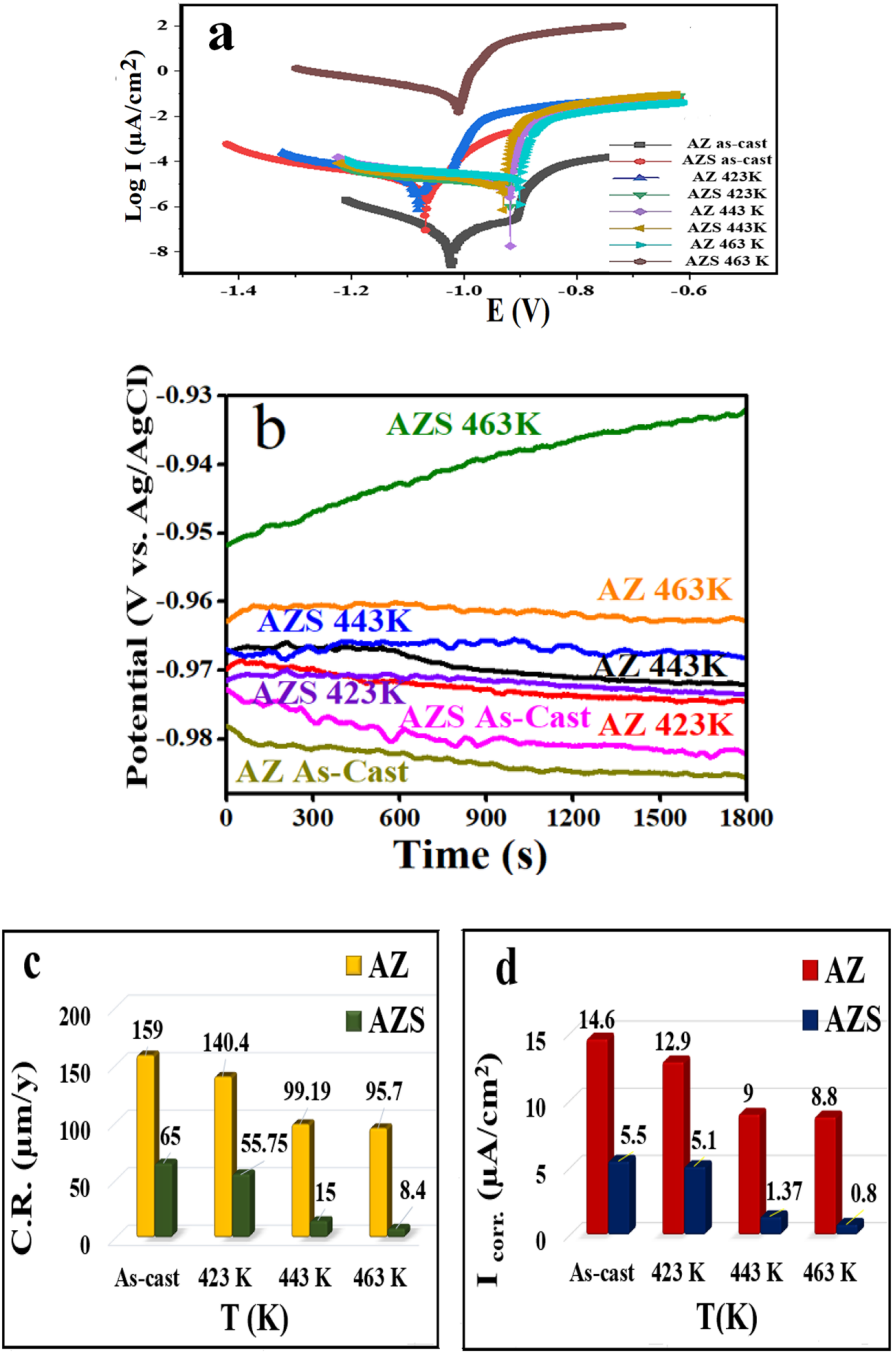
(**a**) Potentiodynamic polarization curves, (**b**) OCP curves of the AZ and AZS in a 3.5% NaCl solution; relation between (**c**) C.R. and (**d**) I_corr._ vs. temperature of the AZ and AZS before and after aging.

The values of the open circuit potential (OCP) of samples AZ and AZS were observed for 2000s after being as-cast and aged at different temperatures, as shown in Fig. 6b. The OCP increased steadily with increasing aging temperature and value-added by adding nano-silica compared with the AZ. The potential of AZ increased from − 0.95181 to − 0.96298 V vs. Ag/AgCl by adding nano-silica. The potential increased from − 0.96694 to − 0.96755 V vs. Ag/AgCl at 423 K, while it increased from − 0.96992 to − 0.97155 V vs. Ag/AgCl at 443 K. The optimal age for AZ and AZS was found to be at 463 K, where the potential shift was from − 0.9728 to − 0.97813 V vs. Ag/AgCl, revealing the impact of nano-silica addition and aging on a positive potential shift by increasing aging. Furthermore, AZS decelerated its potential stability in a 3.5% NaCl solution, which supports the Tafel test.

The study investigated the impact of adding 1% nano-silica to an Al–10 Zn alloy on corrosion attack in a 3.5% NaCl solution at room temperature. The Tafel extrapolation measurements, as shown in Fig. 6a, were used to determine the corrosion parameters and the corrosion rate. The results showed that the AZS corrosion rate (C.R.) was lower than the AZ, and corrosion current density (I_corr._) decreased over the testing period. The Tafel plot showed a drop in E_corr_, I_corr._, and C.R. in as-cast and aged samples, as illustrated in Fig. 6c and d. The research shows that raising the temperature of aging leads to a decrease in I_corr._ for AZ alloy compared to as-cast samples^9^. However, adding nano-silica to Al–10wt%Zn alloy improves corrosion resistance. AZS showed superior corrosion resistance to AZ samples at all aging temperatures. I_corr._ decreases proportionally to material C.R.^36,37^. As illustrated in Fig. 6c,d, there is a direct correlation between the presence of stiff particles (nano-silica) and improved corrosion resistance^24^. The alloy and grain size diameter are related to the improvement of electrochemical and mechanical properties as a result of a uniformly distributed SiO_2_ nanostructure^38–40^. Increasing the aging temperatures for both AZ and AZS inhibits the corrosion process^9^. In electrochemical corrosion testing, a tiny quantity of nano-silica boosted surface area, lowering corrosion sites and delaying C.R. before and after aging. The higher specific surface area of nano-silica could help clarify its corrosion resistance^7^. The aged samples displayed a more uniform distribution of nano-silica in AZ, which limits charge transfer in the composite surface and provides superior corrosion resistance during corrosion testing than AZ. As a result, the sample AZS containing nano-silica has higher corrosion resistance than the other AZ. AZS gives additional stability compared with AZ before and after aging; hence, nano-silica plays a vital role in providing high corrosion protection^24^.

**Figure 7:**
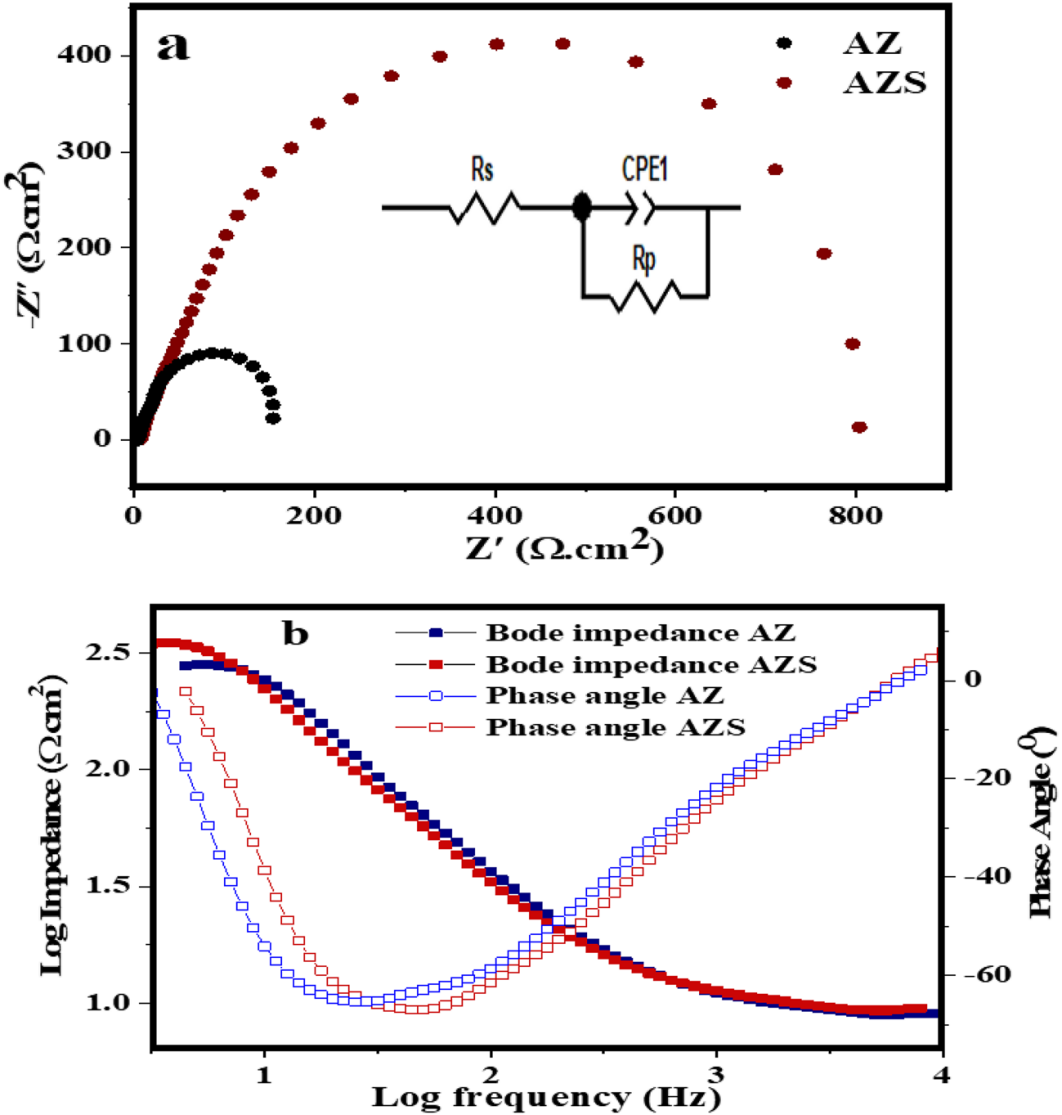
(**a**) Nyquist and the equivalent electrical circuit model, (**b**) Bode impedance, and phase angle plots of AZ and AZS composites at aged temperatures 463 K.

Figure 7a illustrates the corrosion behavior of the AZ alloy and AZS in a NaCl-based solution, according to EIS and the equivalent electrical circuit model. The results show that the AZS has better corrosion resistance than the AZ. Table 5 provides the reference parameters that were obtained from the EIS fitting plots by manual Zview software adjustments for the composites. The dense and stable surface of AZS may provide good protection against corrosive environments, as indicated by the larger capacitive response and greater phase angle (Fig. 7b). The Nyquist graphs are compatible with the phase angle and bode impedance values. As a result, AZ corroded even more than the AZS sample because the corrosion product layer on the AZ surface has a lower density. It was observed that the corrosion resistance effectiveness of AZ and AZS increased with age (423, 443, and 463 K)^9^. The impact of adding nano-silica. This was due to the demand for extremely tiny and smaller particle sizes, as well as a low probability of pitting, cracking, and voiding. The blocking boosted the nanosample's corrosion resistance by making the surface passive and protecting the AZS surface from ionization and dissolution. This result illustrates how the nano-silica addition improves the Al–Zn surface’s corrosion resistance in corrosive environments at room temperature^41^.

**Table 5:** The reference parameters were extracted from the EIS fitting plots for the composites.

Sample	Rs	Rp
AZ	15	186
AZS	11	802

#### Corrosion rate and corrosion current density

The AZS nanocomposite showed superior corrosion resistance when silica nanorods were added to AZ, reducing I_corr_ and increasing surface area^27^. The C.R. is calculated using the following formula:

2 $$\text{C}.\text{R}. =\frac{0.0032 \times \text{ Icorr}\times (\text{M}.\text{W}.) }{\text{n }\times \text{ d}}$$

where C.R. is the corrosion rate (mpy), I_corr_ is the current density of corrosion (A cm^−2^), M.W. represents the corroded material’s molecular weight (g/mol), n is the number of charge transfers across the corrosion process, and d is the density (g cm^−3^)^42^. This behavior was observed at as-cast and after-all aging temperatures, as seen in Fig. 6c and d. The samples exhibited two distinct behaviors: a decrease in C.R. at as-cast temperatures of 423 K and 443 K and an increase in C.R. at 463 K. The addition of nano-silica increased surface area, minimizing corrosion sites and lowering C.R. before and after aging. The treated nanosamples had a more uniform nanostructure distribution^38,39,43^ (Fig. 3) and improved electrochemical characteristics by regulating charge transfer on the composite surface.

#### Surface morphology after corrosion

**Figure 8:**
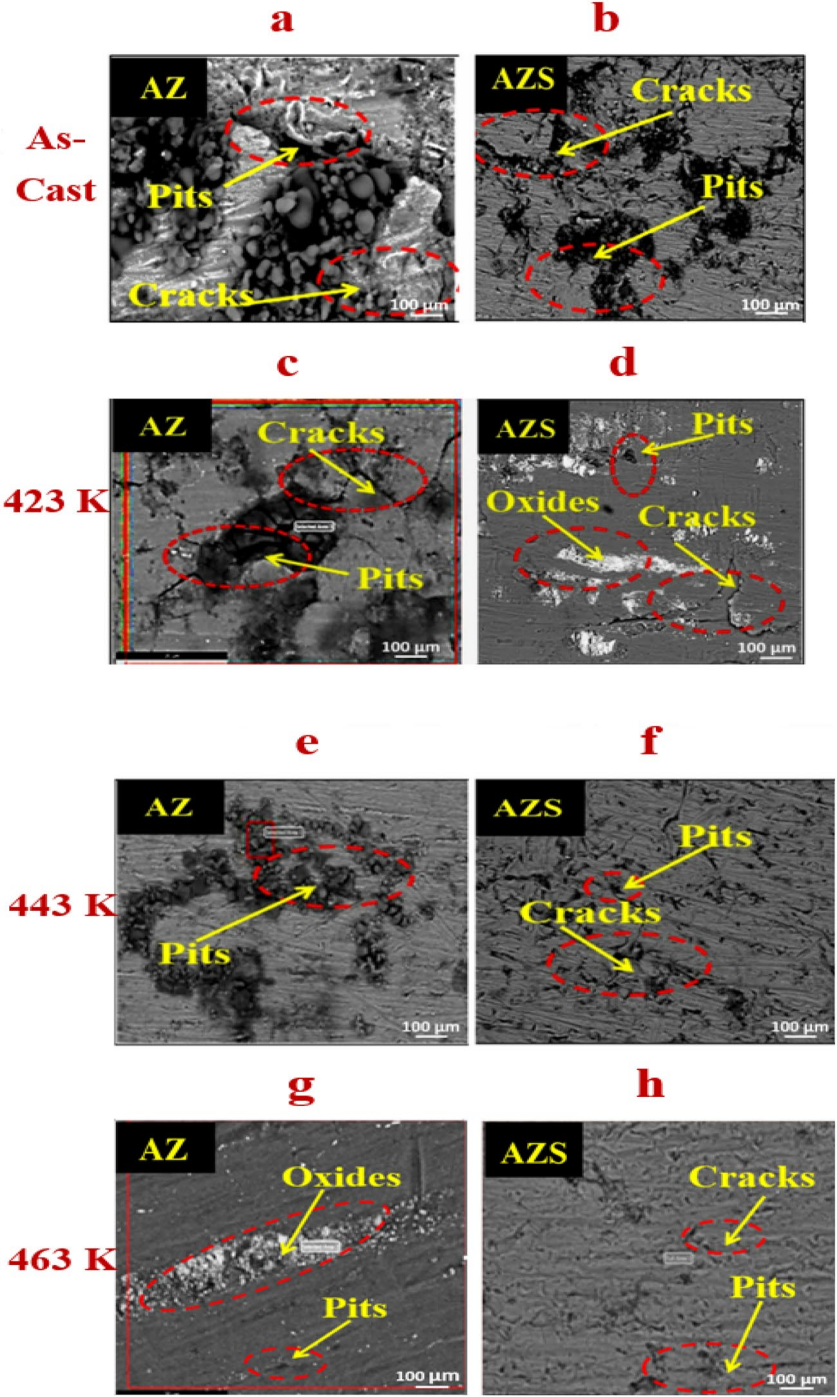
SEM images after the corrosion test before and after different aging temperatures of AZ and AZS with a scale bar of 100 µm.

Figure 8a–h depicts the microstructure SEM micrographs of the AZ and AZS composites obtained from the corroded surface for as-cast and after aging at 423, 443, and 463 K. Aging and reinforcement of NSs can alter the sample surface microstructures, corroborating the C.R. results. Figure 8a,c,e, and g show the AZ surface, whereas Fig. 8b,d,f, and h represent the AZS. The sample’s surfaces corroded by the NaCl solution show irregular pitting, cracks, and ruptured oxide, indicating electrochemical activity regions for AZ and AZS. As a result, AZS has much higher corrosion resistance than AZ, as shown by the C.R. and I_corr_ values for all samples in Fig. 8a and b. After aging at 423, 443, and 463 K, all samples showed a drop in C.R. and I_corr_ as the temperature rose due to a decrease in dislocation pinning activity and, hence, surface area^44^, with a significant rise at the transition temperature at  443 K. The AZS is a superior composite due to the presence of nano-silica distributed on the surface and its greatest surface area (Fig. 8). The perfect dispersion of nano-silica in AZS is without agglomeration, as demonstrated by SEM images. As a result, C.R. is higher than in the other AZS nanocomposites. The drop in C.R. values, Fig. 8a, from as-cast to 463 K for all samples might be due to Zn atom dissolution in the Al matrix, as shown by SEM and EDX of AZ and AZS after 2 h of aging at 463 K. The dissolving procedure reduces dislocation interaction with Zn atoms, slightly increasing grain size while decreasing dislocation density and lattice strain^9^, as shown in Table S1 (Supplementary information) enhancing C.R. However, nanostructures aggregate on the grain boundary when temperature increases. Smaller grain sizes lead to reduced segregation, and unified corrosion occurs. Improving grain refinement lowers corrosion rates^45,46^. Fine grain structures are more corrosion-resistant due to high grain boundary density and accelerated oxide layer conduction. Several studies have revealed that dislocations affect corrosion performance. These results show that aging temperature significantly affects the microstructure and electrochemical properties of the samples. As a consequence, it is observed that increasing the aging temperature reduces the C.R., and nano-silica addition to the Al-10 Zn alloy reduces microcrack energy and nucleation sites and prevents propagation^45,46^.

Finally, the AZS nanocomposite alloy has better corrosion resistance both before and after aging temperatures, as shown in Fig. 8a–h. Nevertheless, compared to the AZ base alloy, it is also more prone to tiny pitting corrosion and cracks with oxide detection. However, when the temperature increased to 463 K, the flawless dispersion of nano-silica in AZS produced a consistent corrosion behavior and a decrease in C.R^9^. The aging process and the addition of silica to the base alloy caused Zn atoms and nano-silica to precipitate during quenching along grain or sub-grain boundaries, potentially further lowering the AZ base alloy's resistance to corrosion.

## Conclusions

In this study, nanostructures were added and analyzed for the Al-10 Zn alloy using XRD, SEM, mechanical, and electrochemical tests. The addition of SiO_2_ nanostructures to the alloy resulted in ultra-grain refinement and increased Vicker's hardness by 13.8%. The nanocomposite AZS was more ductile and stronger than AZ, with high σ_UTS_ values of ~ 79% at 443 k. Aging contributed to the improvement of ductility, tensile strength and plasticity. The addition of silica nanorods improved electrochemical behavior, decreasing corrosion behavior and enhancing the corrosion rate by 89.4% compared to the base alloy. Heat treatments were effective in modifying the microstructure (Supplementary Information).

### Supplementary Information

Supplementary Information.

## Data Availability

Data sets generated during the current study are available from the corresponding author on reasonable request.
