# Supplementary material for: Corrosion suppression and strengthening of the Al-10Zn alloy by adding silica nanorods
Source: Sci Rep. 2024 Jul 8;14:15644. doi: 10.1038/s41598-024-64323-x (PMC11231230; doi:10.1038/s41598-024-64323-x)
Supplement: Supplementary file 1 — Supplementary Information. [file 41598_2024_64323_MOESM1_ESM.docx]

**Corrosion suppression and strengthening of the Al-10Zn alloy by adding silica nanorods**
Eman AbdElRhiem^1,2^*, Yosry F. Barakat^1^, Shereen M. Abdelaziz^2^, M. M. Mostafa^2^, R. H. Nada^2^ and Saad G. Mohamed^1^*

^1^ Mining and Metallurgy Engineering Department, Tabbin Institute for Metallurgical Studies (TIMS), Tabbin, Helwan 109, Cairo 11421, Egypt. ^2^ Physics Department, Faculty of Education, Ain Shams University, Heliopolis 11771, Roxy, P.O. Box 5101, Cairo, Egypt.

*Corresponding Authors: Eman AbdElRhiem ([eman_tims@yahoo.com](mailto:eman_tims@yahoo.com)), and S. G. Mohamed (saadmohamed@tims.gov.eg)

**XRD analysis**


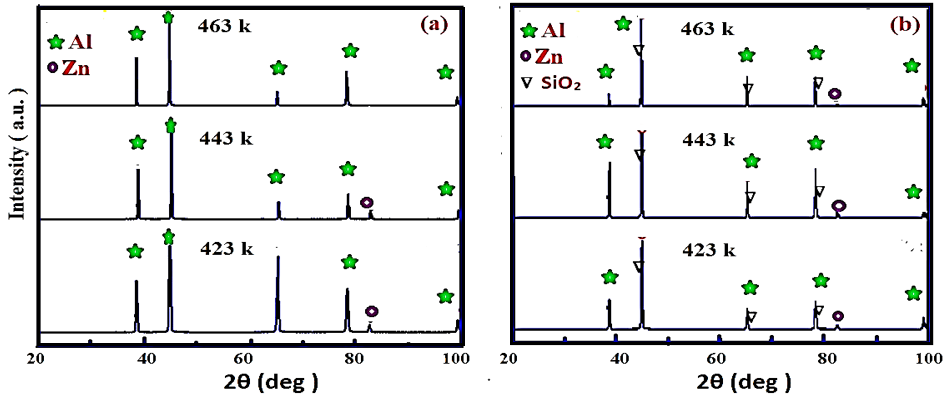


**Figure S1.** XRD patterns of (**a**) **AZ** and (**b**) **AZS** at different aging temperatures.

The morphological analysis performed by OM, SEM, and EDX is supported by the XRD study. The XRD patterns of the AZ and AZS samples at different aging temperatures (423, 443, and 463 k) are displayed in Fig. S1(a, b). The AlZn anorthic structure with a single-phase homogeneity distribution, in which Zn is completely dissolved in the Al matrix at 463 K, was shown by the XRD patterns of the AZ (base alloy) and AZS composite alloy. The SiO_2_ (NSs) addition to the Al-10 Zn alloy appears to have formed different SiO_2_ peaks, as seen in Figure 2(d).
XRD data can be used to compute the average crystallite size, D, of the Al-rich phase using Williamson-Hall^’^s formula ^[9]^.

β $\frac{cos \theta}{\lambda}$ = $\frac{1}{D}$ + 2 ε $\frac{sin \theta}{\lambda}$ (1)

Where the wavelength of the radiation **λ**, the Bragg angle **θ**, and$\beta$, the radian value of the full width at half-maximum (FWHM).

The dislocation density (ρ_d_) can be calculated using the following expression ^[9].^

$\rho_{d}=14.4 \frac{ɛ^{2}}{b^{2}}$ (2)

**Table S1.** Lists the average crystallite size, D and the dislocation density ρ_d ,_ as a function of aging temperature for AZ and AZS samples.

| Sample | **Average crystallite size, D×10^-6^**  **(m)** | | | **dislocation density (ρ_d_) × 10^9^ /m^2^** | | | **Lattice strain (ɛ)**  **× 10^-4^** | | |
| --- | --- | --- | --- | --- | --- | --- | --- | --- | --- |
|  | 423 K | 443 K | 463 K | 423 K | 443 K | 463 K | 423 K | 443 K | 463 K |
| **AZ** | 102 | 77 | 90 | 2.6 | 4. 3 | 1.5 | 0.10 | 0.12 | 0.05 |
| **AZS** | 80 | 59 | 65 | 5 | 7.3 | 3.33 | 0.15 | 0.16 | 0.08 |
